# Infectious Complications, Healthcare Resource Use, and Medical Costs Associated with Delays in Percutaneous Nephrolithotomy Among Patients with Stone Disease and Ureteral Stent Placement

**DOI:** 10.1089/end.2022.0489

**Published:** 2023-06-06

**Authors:** Naeem Bhojani, Rutugandha Paranjpe, Benjamin Cutone, Brian H. Eisner

**Affiliations:** ^1^ Division of Urology, Centre Hospitalier de l'Université de Montréal, Montreal, QC, Canada.; ^2^ Division of Urology, Boston Scientific, Marlborough, Massachusetts, USA.; ^3^ Harvard Medical School, Massachusetts General Hospital, Boston, Massachusetts, USA.

**Keywords:** kidney stones, ureteral stent, percutaneous nephrolithotomy, health care resource utilization, cost

## Abstract

**Purpose:**

The relationship between ureteral stent duration before percutaneous nephrolithotomy (PCNL) and infectious complications, admissions, imaging, and medical costs was evaluated.

**Materials and Methods:**

Patients who underwent PCNL within 6 months of ureteral stent placement were identified from commercial claims, categorized by time to treatment (0–30, 31–60, and >60 days), and followed 1-month post-PCNL. The effect of delayed treatment on inpatient admissions, infectious complications (pyelonephritis/sepsis), and imaging utilization was evaluated with logistic regression. A generalized linear model evaluated the effect of delayed treatment on medical costs.

**Results:**

Among 564 patients with PCNL and meeting the inclusion criteria (mean age 50; 55% female; 45% from South), mean (standard deviation) time to surgery was 48.8 (41.8) days. Less than half (44.3%; *n* = 250) underwent PCNL within 30 days of ureteral stent placement, 27.0% (*n* = 152) between 31 and 60 days, and 28.7% (*n* = 162) >60 days. Time to PCNL was significantly associated with inpatient admissions (>60 *vs* ≤30 days odds ratio [OR] 1.97, 95% confidence interval [CI] 1.29–3.01, *p* = 0.0016), infectious complications (>60 *vs* ≤30 days OR 2.43, 95% CI 1.55–3.81, *p* = 0.0001), imaging utilization (31–60 *vs* ≤30 days OR 1.56, 95% CI 1.02–2.38, *p* = 0.0383; >60 *vs* ≤30 days OR 2.01, 95% CI 1.31–3.06, *p* = 0.0012), and medical costs (31–60 *vs* ≤30 days OR 1.27, 95% CI 1.08–1.49, *p* = 0.0048; >60 *vs* ≤30 days OR 1.46, 95% CI 1.24–1.71, *p* < 0.0001).

**Conclusions:**

Compared with PCNL within 30 days, patients undergoing PCNL >30 days after ureteral stent placement had increased likelihood of infectious complications, resource use, and medical costs. These results may inform health care resource utilization and PCNL prioritization.

## Introduction

The incidence and prevalence of kidney stones are increasing globally across sexes, races, and ages,^1^ affecting ∼8%–12% of the world population.^2–6^ Percutaneous nephrolithotomy (PCNL) is the standard of care for large renal stones, resulting in the highest stone free rates and lowest retreatment rates when compared with other minimally invasive treatments.^7–9^ PCNL may be preceded by ureteral stent placement, either for emergent drainage in cases of obstruction, infection, and renal insufficiency or as a temporary measure for pain relief when primary treatment is not available or not possible because of other medical conditions.^10,11^ PCNL operative techniques and technologies have constantly evolved since its introduction in 1976, increasing the success rates and decreasing complications and morbidity.^12^ However, the rate of PCNL has remained stable over the years as this is the most effective and efficient way to treat larger stones within the kidney.^8,9^

The preoperative placement of ureteric stent is sometimes required as stated above, however, it is well recognized that ureteral stents may be a source of significant morbidity.^13–15^ Unwanted commonly reported effects of ureteric stents include flank pain, storage symptoms, hematuria, and dysuria.^16^ There is also the more concerning hazard of increased risk of urinary tract infection (UTI).^16^ The introduction of a foreign body into the patient's urinary system increases the risk of bacterial colonization and bacteriuria, beginning when the stent is inserted and progressing with stent dwelling time.^17^ Patients with longer indwelling ureteral stent duration before definitive stone treatment may have worse outcomes than those with shorter ureteral stent duration.^16^ The current study sought to evaluate the effects of ureteral stent duration before PCNL on infectious complications, health care resource utilization (HCRU), and medical costs.

## Materials and Methods

### Data source

This retrospective observational cohort study used United States (US) administrative claims data from the IBM MarketScan Commercial Claims and Encounters databases from July 1, 2014, to June 30, 2020. These databases include enrollment information, demographics, inpatient and outpatient medical, and outpatient pharmacy claims data, derived from more than 300 large, self-insured US employers and over 25 US health plans. Commercial data represent individuals ≤65 years of age (primary insured, spouse, or dependent). Ethics approval from an Institutional Review Board and Informed Consent were not required for this study given that the data were from an anonymous, deidentified, administrative claims database compliant with the Health Insurance Portability and Accountability Act of 1996.

### Patient population

Patients with a stone diagnosis and a code indicative of ureteral stent placement (Current Procedural Terminology code 52332 or International Classification of Diseases, 9th Revision [ICD-9] code 57.32) from January 1, 2015, to November 30, 2019 were considered for inclusion in the study. The date of ureteral stent placement was defined as the “index date.” Patients were required to have undergone follow-up PCNL stone surgery within 6 months postindex (based on clinical expertise), continuous enrollment 6 months before index (i.e., the “baseline” period) until 1 month after the date of PCNL stone surgery and be ≥18 years of age. Patients with a stone procedure (ureteroscopy [URS], shockwave lithotripsy, or PCNL) or ureteral stent placement 90 days before the index date were excluded from the study.

**FIG. 1.**
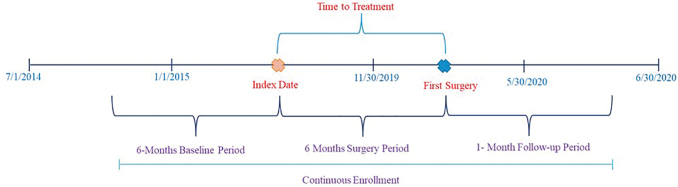
Study design schematic. Color images are available online.

Also, patients with infections (i.e., pyelonephritis or sepsis) on the index date were excluded. Patients with infections on the index date could have biased the results and hence were excluded. Finally, patients who had health care utilization costs ≤$0 were also excluded as these were presumably due to missing data or clerical inaccuracies in the administrative database.^18–20^ Figure 1 presents the study design.

### Study measures

#### Baseline demographic and clinical characteristics

Patient demographic and clinical characteristics that were evaluated included age, sex, geographic region, and patient comorbidities. Age, sex, and geographic region were measured on the index date. Patients from the Northeast, Midwest, and West were categorized as “other” and compared against the “south” region due to sample size limitations. Baseline (i.e., period 6-months before the index date) comorbidity (i.e., comorbid conditions present before the index date) was assessed using the Elixhauser Comorbidity Score, an aggregate measure of comorbidity created by using 31 dimensions associated with chronic disease (e.g., heart disease, cancer), and overall health conditions. Prior research has shown that increasing Elixhauser Comorbidity Scores are associated with increased health care utilization and greater risk of mortality.^21,22^

#### Infectious complications

The complications that were evaluated were infections (i.e., pyelonephritis, sepsis, cystitis, and UTI) associated with a primary diagnosis that occurred during the study period. The outcome period entailed 1 day postindex (i.e., ureteral stent placement) to 1-month post-PCNL.

#### HCRU and costs

Health care resource use evaluated during the study period included the number of inpatient admissions associated with a primary diagnosis of stone disease or infectious complications. Imaging procedures (i.e., CT scans, ultrasounds, and x-rays) associated with a primary diagnosis of stone disease were also evaluated. Finally, medical costs associated with a primary diagnosis of stone disease or infectious complications were evaluated during the study period and were adjusted to 2020 US dollars using the Consumer Price Index. The outcome period entailed 1 day postindex (i.e., ureteral stent placement) to 1-month post-PCNL.

### Statistical analyses

All study variables were analyzed descriptively. Counts and proportions (categorical variables) and means and standard deviations (SDs) (continuous variables) were evaluated. The time to PCNL treatment was calculated and patients were categorized into groups: 0–30, 31–60, and >60 days. Group differences were assessed using chi-square tests for categorical variables and one-way analysis of variance (ANOVA) for continuous variables. Logistic regression was conducted to evaluate the effect of delayed treatment on bivariate outcome variables (i.e., infectious complications, inpatient admissions, and imaging) controlling for age, sex, geographic region, Elixhauser Comorbidity Score, and the presence of specific patient comorbidities (i.e., diabetes and hyperlipidemia as these were identified *a priori* as potential risk factors associated with infection and health care resource use^23,24^).

A generalized linear model with gamma distribution and log link was used to evaluate the effect of delayed treatment on medical costs. All analyses were performed using the Instant Health Data software (Panalgo, Boston, MA) and R, version 3.2.1 (R Foundation for Statistical Computing, Vienna, Austria) at *a priori* significance of 0.05.

## Results

### Patient selection

**FIG. 2.**
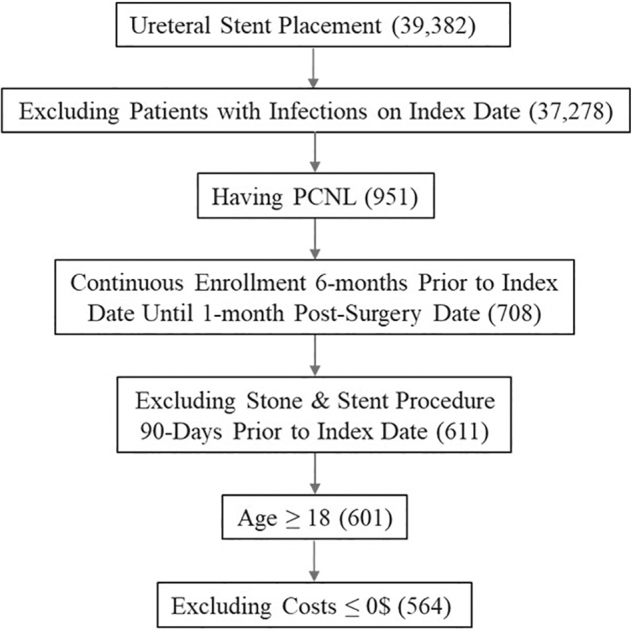
Patient selection using the inclusion and exclusion criteria. PCNL = percutaneous nephrolithotomy.

A total of 39,382 patients from the IBM MarketScan Commercial Claims and Encounters databases had a diagnosis of stone disease and evidence of the placement of a ureteral stent from January 1, 2015, to November 30, 2019. Of those 39,382 patients, 951 underwent PCNL. After applying the inclusion and exclusion criteria, 564 patients were selected (Fig. 2).

### Time to PCNL

The mean (SD) time to PCNL was 48.8 (41.8) days. Less than half of the patients (44.3%) underwent PCNL within 30 days of ureteral stent placement, 27.0% underwent PCNL between 31 and 60 days of ureteral stent placement, and 28.7% underwent PCNL >60 days poststent placement.

### Patient baseline demographic and clinical characteristics

Mean patient age between the groups ranged from 48.3 (±11.6) to 51.5 (±10.2) years old (Table 1). Similarly, between 52.5% and 60.5% of the patients were female, and 44.2%–46.6% of the patients were from the South Census region of the US. Diabetes ranged between 18.4% and 23.5% and hyperlipidemia ranged from 19.7% to 31.5% between the groups. Age, mean Elixhauser Comorbidity Score, and the proportions of patients with hyperlipidemia increased among patients who underwent PCNL from 0–30 days to >60 days. The proportions of patients with diabetes increased with increasing time to PCNL.

**Table 1.** Baseline Demographic and Clinical Characteristics of Patients Undergoing Percutaneous Nephrolithotomy After Ureteral Stent Placement for Stone Disease

Variable	0–30 days* n* = 250	31–60 days* n* = 152	>60 days* n* = 162	*p*
Age; mean (SD)	50.1 (10.4)	48.3 (11.6)	51.5 (10.2)	**0.0319**
Sex				0.3049
Female	135 (54.0%)	92 (60.5%)	85 (52.5%)	
Male	115 (46.0%)	60 (39.5%)	77 (47.5%)	
Region				0.9118
South	107 (45.0%)	68 (46.6%)	68 (44.2%)	
Other (Northeast, Midwest, West)	131 (55.0%)	78 (53.4%)	86 (55.8%)	
Elixhauser Comorbidity Score; mean (SD)	1.80 (1.75)	1.68 (2.07)	2.49 (2.62)	**0.0008**
Comorbidities				
Diabetes	46 (18.4%)	29 (19.1%)	38 (23.5%)	0.4300
Hyperlipidemia	74 (29.6%)	30 (19.7%)	51 (31.5%)	**0.0400**

The demographics were calculated on the index date while the Elixhauser Comorbidity Score and the other comorbidities were calculated in the baseline period.

Bolded *p*-values denote statistical significance at *p* < 0.05.

SD = standard deviation.

### Resource use and costs

Compared to patients who underwent PCNL within 30 days of ureteral stent placement, PCNL between 31 and 60 days after ureteral stent placement was significantly associated with increased imaging utilization (odds ratio [OR] 1.56, 95% confidence interval [CI] 1.02–2.38, *p* = 0.0383) and medical costs (OR 1.27, 95% CI 1.08–1.49, *p* = 0.0048).

Compared to patients who underwent PCNL within 30 days of ureteral stent placement, PCNL >60 days after ureteral stent placement was significantly associated with increased inpatient admissions (OR 1.97, 95% CI 1.29–3.01, *p* = 0.0016), occurrence of infectious complications (OR 2.43, 95% CI 1.55–3.81, *p* = 0.0001), imaging utilization (OR 2.01, 95% CI 1.31–3.06, *p* = 0.0012), and medical costs (OR 1.46, 95% CI 1.24–1.71, *p* < 0.0001) (Table 2).

**Table 2.** Health Care Resource Utilization and Medical Costs by Time to Percutaneous Nephrolithotomy

Outcome	OR	Lower CI	Upper CI	*p*
Inpatient admissions				
31–60 days *vs* ≤30 days	1.22	0.80	1.88	0.3592
>60 days *vs* ≤30 days	1.97	1.29	3.01	**0.0016**
Infectious complications				
31–60 days *vs* ≤30 days	1.46	0.92	2.31	0.1116
>60 days *vs* ≤30 days	2.43	1.55	3.81	**0.0001**
Imaging				
31–60 days *vs* ≤30 days	1.56	1.02	2.38	**0.0383**
>60 days *vs* ≤30 days	2.01	1.31	3.06	**0.0012**
Medical costs				
31–60 days *vs* ≤30 days	1.27	1.08	1.49	**0.0048**
>60 days *vs* ≤30 days	1.46	1.24	1.71	**<0.0001**

Bolded *p*-values denote statistical significance at *p* < 0.05.

CI = confidence interval; OR = odds ratio.

Finally, among the PCNL cohort, average medical costs increased as the time to PCNL increased. The average medical costs among patients undergoing PCNL >60 days after ureteral stent placement were higher compared to patients undergoing PCNL between 31 and 60 days and patients undergoing PCNL within 30 days ($43,215 *vs* $36,041 and $29,313, respectively; *p* < 0.0001).

## Discussion

There is a paucity of data for large populations of patients with stone disease and ureteral stent placement who subsequently undergo PCNL. To our knowledge, this is the first study evaluating the treatment patterns among patients receiving a ureteral stent and then subsequently undergoing PCNL. Study findings showed that <50% of the patients underwent PCNL within 30 days after ureteral stent placement, and nearly 30% underwent PCNL more than 60 days after ureteral stent placement. The time between ureteral stent placement and PCNL was greater among older patients and patients with greater comorbidity.

Our study demonstrated that longer stent duration was associated with increased rate of infectious complications as well as increased inpatient admissions. Compared to patients who underwent PCNL within 30 days of ureteral stent placement, those who underwent PCNL 60 days after stent placement or more experienced a twofold increased rate of inpatient admissions and a nearly 2.5-fold rate of infectious complications. This study is also the first study to evaluate the clinical and economic effects of ureteral stent duration among patients subsequently undergoing PCNL. Greater time to PCNL was associated with significantly greater inpatient admissions, infectious complications, imaging, and medical costs.

Average medical costs increased from $29,313 when patients underwent PCNL within 30 days of ureteral stent placement to $36,041 when patients underwent PCNL between 31 and 60 days after ureteral stent placement, and to $43,215 when patients underwent PCNL more than 60 days after ureteral stent placement. Hence, the study findings suggest potential clinical and economic advantages of not delaying PCNL after ureteral stenting.

As mentioned, to our knowledge, there are no previously published studies evaluating the effects of ureteral stent duration among patients subsequently undergoing PCNL. The advantages observed in this study are in alignment with a previously published retrospective cohort study of kidney stone patients with ureteral stenting before undergoing URS and not PCNL. Nevo and colleagues^16^ evaluated the association between ureteral stent dwelling time and sepsis after undergoing URS (*n* = 256) and found that sepsis rates after ureteral stent dwelling times of 1, 2, 3, and >3 months were 1.0%, 4.9%, 5.5%, and 9.2%, respectively. Multivariable analysis conducted by the authors confirmed that ureteral stent dwelling time was significantly associated with post-URS sepsis after controlling for covariates.

In addition, several additional studies have demonstrated similar findings in URS—longer stent indwelling times between initial stent placement and formal URS treatment for stones is associated with increasing complications.^25^ While other studies have failed to demonstrate this finding,^26,27^ the discordance between these studies may be due to the inclusion of distinct patient populations with different risks in terms of postoperative infection.

A limitation of this study is the use of administrative claims data, which are not collected specifically for research purposes. Administrative claims may have underreported or missing data, clerical inaccuracies, recording bias secondary to financial incentives, and temporal changes in billing codes.^18–20^ Clinical data which could potentially affect time to treatment such as type of stone, recurrent *vs* new stone former, staghorn *vs* midsized stone suitable for mini PCNL, placement of stents for contralateral or ipsilateral stones, was not available. Further, data on indications for stent insertion, and type of PCNL (conventional or mini PCNL) was not available. Also, the findings from the IBM MarketScan Commercial Claims and Encounters databases may not be generalizable to other patients with stone disease, ureteral stent placement, and stone surgery, particularly patients without commercial health insurance (such as Medicare) or patients in other countries.

Finally, the PCNL follow-up period included data from March 2020-May 2020, which includes the first surge of COVID-19 in the United States. This might have potentially delayed care. Despite these limitations, this study provides contemporary and valuable information that is currently deficient in the literature regarding the potential impact of delaying PCNL stone surgery after ureteral stent placement. Finally, while a delay in PCNL treatment after stent insertion could also reflect a problem with the patient (severe sepsis/UTI/medical issue), future studies should evaluate precautionary measures to prevent any adverse events that could plausibly delay PCNL treatment.

## Conclusions

In this large retrospective database analysis of commercially insured patients in the United States, patients who underwent PCNL >30 days after ureteral stent placement had a greater risk of imaging utilization and increased medical costs. Patients who underwent PCNL >60 days after ureteral stent placement had a greater risk of infectious complications, inpatient admissions, imaging utilization, and increased medical costs compared with patients who underwent PCNL ≤30 days. Earlier PCNL after the placement of a ureteral stent may reduce overall HCRU and cost of patient care.

## Abbreviations Used

ANOVA: analysis of variance
CI: confidence interval
CT: computed tomography
HCRU: health care resource utilization
ICD-9/10-CM: International Classification of Diseases 9^th^/10^th^ Edition, Clinical Modification
OR: odds ratio
PCNL: percutaneous nephrolithotomy
SD: standard deviation
URS: ureteroscopy
UTI: urinary tract infection
